# Single-photon multi-ports router based on the coupled cavity optomechanical system

**DOI:** 10.1038/srep39343

**Published:** 2016-12-22

**Authors:** Xun Li, Wen-Zhao Zhang, Biao Xiong, Ling Zhou

**Affiliations:** 1 School of Physics and Optoelectronic Technology, Dalian University of Technology, Dalian, 116024, People’s Republic of China

## Abstract

A scheme of single-photon multi-port router is put forward by coupling two optomechanical cavities with waveguides. It is shown that the coupled two optomechanical cavities can exhibit photon blockade effect, which is generated from interference of three mode interaction. A single-photon travel along the system is calculated. The results show that the single photon can be controlled in the multi-port system because of the radiation pressure, which should be useful for constructing quantum network.

Quantum router to combine quantum channels with quantum nodes can create a quantum network so as to distribute quantum information. Recently, many theoretical proposals and experimental demonstrations of a quantum router have been carried out in various systems. One-dimensional single-photon efficient router in cavity QED system has been realized^1^. By employing the EIT effect to guarantee single photon transportation, Io-Chun Hoi *et al*.^2^ achieved a single-photon router in microwave regime. Different kinds of schemes of multi-port router have also been proposed, for instance cyclic three-level Δ-type atom system is used to route photon into two coupled cavity arrays^3^,^4^. Linear-optical system^5^,^6^,^7^,^8^ also is regarded as a rational candidate of quantum router because of easy-control and easy-achieve property despite lack of capacity of routing single photon. Recently, people focus their vision on mesoscopic scale devices on account of its nonlinearity and controllability, such as optomechanical system^9^ and cavity electromechanical system^10^.

Photon-blockade phenomenon resulted from nonlinearity allows only one photon existence, and the second photon will be prohibited, which can be used to generate single photon source or to ensure a single photon processing. Cavity optomechanical systems, besides its potential application in detecting gravity waves^11^,^12^, in studying quantum-to-classical transitions^13^, in performing high precision measurements^14^,^15^,^16^,^17^, in entanglement generation^18^,^19^,^20^ and preservation^21^ and in processing quantum information^16^,^22^,^23^,^24^,^25^, are of nonlinearity^26^,^27^,^28^,^29^,^30^,^31^,^32^. But this nonlinear strength proportional to *g*^2^/*ω*_*m*_ is limited by the condition *g* (the coupling strength of radiation pressure) less than *ω*_*m*_ (the frequency of the mechanical oscillator), therefore, a lot of effort is devoted to enhance the nonlinearity, for instance, adding atoms^33^, introducing quantum dot^34^, using coupled cavity optomechanical system^27^ and employing three-mode mixing to generate effective photon blockade^35^.

In this paper, we put forward a scheme by coupling two cavity optomechanical system. We show that our system can be effectively equal to three-mode interaction^35^ and can exhibit photon blockade. Then we construct four output ports by coupling wave guide to the two-cavity-optomechanical system. Our research show that our system can work as multiple output ports router under the assistant of mechanical mode, which provide a potential application for the cavity optomechanical system in multiple router.

## Results

In this part, we introduce our model, illustrate the photon-blockade effect of this two-cavity-optomechanical waveguide coupled system and study the transport of photons of waveguide under photon-blockade condition.

### Model and effective interaction

**Figure 1:**
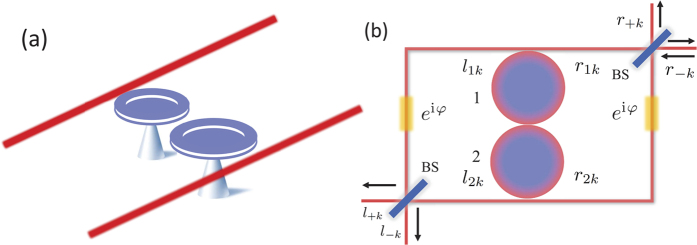
Schematic configuration of the single-photon router. (**a**) The two toroidal cavities with mechanical modes coupling to waveguide. (**b**) The four ports router with quasi-mode. The router consist of optomechanics as a single photon source, fibers, phase delayer with delay phase 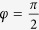 and beam splitters to change photon from normal mode to quasi-mode.

We consider the two optomechanical cavities coupled with hopping coefficient *J*, and the two optomechanical cavities are side-coupled to the fibers respectively. The configuration of the system is shown in Fig. 1a, which is similar with ref. 36 where they utilized the two coupled whispering-gallery-mode (WGM) microtoroids coupled to two tapered fibers to experimentally realize parity–time-symmetric optics, but the mechanical modes are ignored. Taking the mechanical modes into consideration, we write the Hamiltonian as


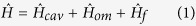


with


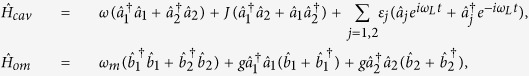


where 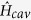 describes the free energy of the cavity, 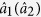 and 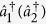 represent the annihilation and creation operators of cavity modes with the same frequency *ω*, and the two cavities are pumping with classical field with frequency *ω*_*L*_ and intensity *ε*_1_, *ε*_2_. 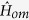 represents the energy of the two mechanical oscillators with frequency *ω*_*m*_ and their coupling with the cavity fields induced by radiation pressure, where the 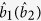 and 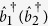 are annihilation and creation operators of mechanical oscillators, *g* is coupling between first (second) cavity field and first (second) mechanical oscillator. The Hamiltonian 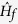 in Eq. (1) can be written as


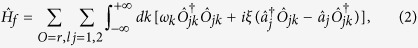


which expresses the two cavity fields coupling with the fibers, where 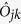 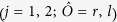 and *ω*_*k*_ represent annihilation operators and frequency of the fibers with wave number *k*, and *ξ* is the strength of coupling. In the frame rotating with 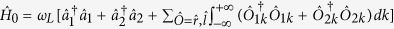, we have


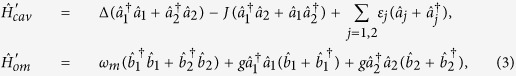


where Δ = *ω*−*ω*_*L*_, and


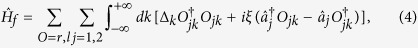


where Δ_*k*_ = *ω*_*k*_−*ω*_*L*_. Now, we introduce the operators


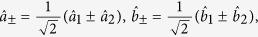


The Hamiltonian 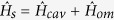 is of the form


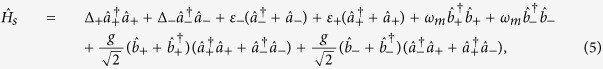


where 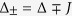, 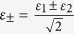. For the fiber, we define


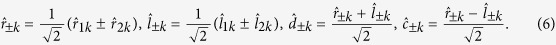


Thus, 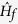 can be rewritten as


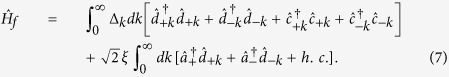


We see that the cavity modes are decoupled with the fiber mode 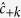 and 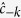. We switch into the picture rotating with 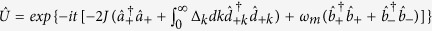, i.e., employing the relation 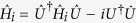, we can rewrite Eq. (5). Considering the condition 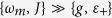 and choosing parameters *ω*_*m*_ = 2*J*, we have the Hamiltonian


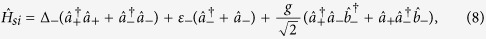


and


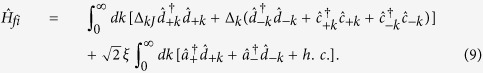


where Δ_*kJ*_ = Δ_*k*_ + 2*J*. Due to rotating-wave approximation, the terms 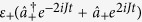, 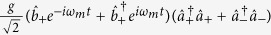 and 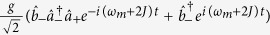 with high frequency oscillation are ignored. The Hamiltonian Eq. (8) indicate the three-body interaction between cavities and the oscillator, which is exact the same with ref. 35 where the nonlinearity has been analyzed. In a single cavity optomechanical system, the effective photon-photon interactions *g*^2^/*ω*_*m*_ is suppressed by the condition that the mechanical frequency is much larger than the coupling *g*, i.e., 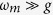, while the three-body interaction (8) has its advantage^35^ that photons in the two optical modes can be resonantly exchanged by absorbing or emitting a phonon via three-mode mixing; therefore, the restraint 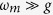 can be overcome. Since our system can be simplified as^35^, one can see that the nonlinearity should be exist and does not restrict by the condition 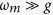. Most importantly, the Hamiltonian Eqs (8) and (9) exhibit clearly the conversion between the quasi-mode between 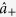 and 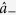 under the witness of 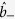 so that we can realize the exchange between 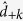 and 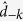. Therefore, with the interaction, we can potentially realize four ports router.

### Photon Blockade

Now we first investigate the nonlinearity of the photons within the cavity. The dynamics of the system obeys the master equation


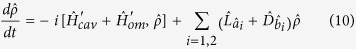


where Lindblad 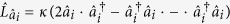 with zero thermal photon and dissipation rate *κ*, 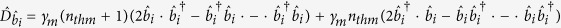 with thermal photon *n*_*thm*_ and dissipation rate *γ*_*m*_, *i* = 1, 2. The fibers can be considered as a part of environment of the cavity modes with the interaction (4), thus, the interaction between the cavities and the fibers can be reduced to the term 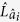. Similarly, the interaction (9) also can be reduced into Lindblad form. Because of the larger frequency difference (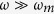), the cavity fields can be treated as in environment with zero thermal photon while for the mechanical oscillators they are involved in thermal reservoir. To characterize the nonlinearity of optical modes, we employ the equal-time second-order correlation functions


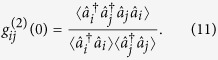


For *i* = *j*, the function 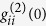 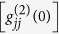 denotes the self-correlation, and 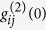 (*i* ≠ *j*) express the cross-correlation. If the correlation function 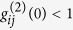 we say the photon anti-bunching, and the limit 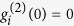 corresponds to the thorough photon blockade effect, which means that only one photon can exist, and the another photon will be blockaded.

**Figure 2:**
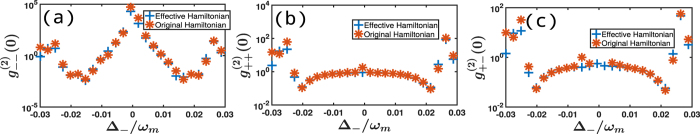
(**a**) Plot a relation between correlation function 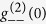 and detuning Δ_−_, blue dot for solve master equation with effective Hamiltonian red dot for original Hamiltonian. (**b**) Correction *g*^(2)^(0) of *a*_+_ as function of Δ_−_. (**c**) Cross correlation function 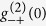 versus detuning Δ_−_. Other parameters are *J* = 2*ω*_*m*_, *g* = 0.03*ω*_*m*_, *κ* = 10^−3^*ω*_*m*_, *n*_*mth*_ = 0.2, *γ*_*m*_ = *κ*/200, *ε*_1_ = 1.1 × 10^−4^*ω*_*m*_, *ε*_2_ = −*ε*_1_.

Now, we show the nonlinearity by comparing the numerically solution of the master equation Eq. (10) with that 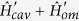 are substituted with effective Hamiltonian Eq. (8) where the subscripts *i* = 1, 2 for the superoperators 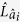 and 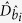 are easily changed to *i* = −, + because we assume the two cavity modes as well as mechanical modes with equal decay rate respectively. As shown in Fig. 2, we see that the solution of master equation with the effective Hamiltonian coincides with that of master equation with original Hamiltonian, which show that the effective Hamiltonian method is reliable. We will employ the effective Hamiltonian Eq. (8) in the calculation of the photon router procession. More importantly, we observe that 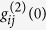 (*i*, *j* = −, +) achieves their minimum values around 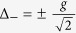, which means that the system can suppress the simultaneous two-photon creations in any of the mode 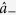 and 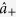, especially the cross mode between 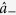 and 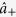. That is to say, in the coupled two cavity optomechanical system, there is most possible only one photon existence. Thus, the property can be potentially used as a single photon router if we can control it. The photon-blockade is resulted from three-body interactions that lead to destructive interference of optical modes. The conclusion is also obtained in ref. 35 where the destructive interference is analyzed with eigenstate of the Hamiltonian Eq. (8). The three-body interaction is still dependent on the coupling *g* see Eq. (8), therefore the strong coupling strength is still welcome. But the nonlinearity is not proportional to 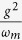, which means that the nonlinearity is not limited by the condition 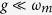.

### Single-photon router

Quantum router is a hinge device for large-scale network communications. How to design quantum router arouse a lot of interests^1^,^2^,^3^,^4^,^5^,^9^,^10^. To satisfy the requirements of quantum information, a single-photon quantum router will be demanded. Photon blockade effect is an effective method to realize the single-photon router. As we have shown in the Fig. 2, there is a good photon blockade phenomenon in this optomechanical system. We can reasonably assume that the device is only allow a single photon transport. Therefore we will only consider a single excitation in the system.

Now, we employ the two coupled optomechanical cavities to couple to two waveguide (CRW) shown in Fig. 1b. In order to employ the quasi-mode, we introduce medium as phase shifter and beam splitters to generate the quasi-mode. One can easy deduce that the four outputs will satisfy the relation Eq. (6). We now calculate the photon number of the four ports. Under the Hamiltonian Eqs (8) and (9), the basis is denoted as 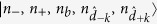, thus we can write the wave function with only a single excitation as


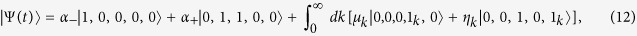


In terms of the left- and right-propagation modes, if we assume a photon packet is incident onto the cavity from the port 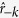, i.e., 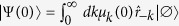, a wave packet with a Lorentzian spectrum 
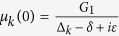
, where *ε* and *G*_1_ are the linewidth and normalization coefficient of Lorentzian spectrum. The wave function obey Schrodinger equation with Hamiltonian 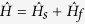. In the long-time limit,we can find the solution of wave function


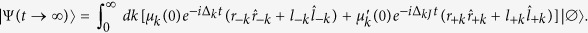


The details of calculation can be found in part methods. Therefore, the output photon number of the four ports are obtained as


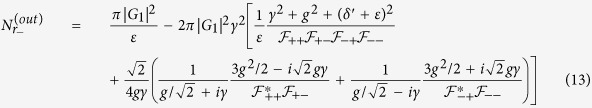



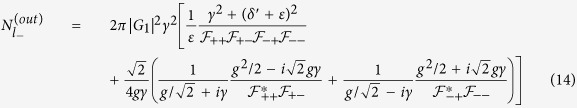



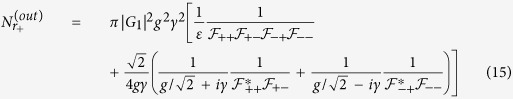



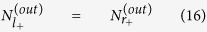


with 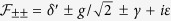, where *δ*′ = *δ*−Δ_−_ and *γ* = 2*πξ*^2^. The detail can be seen in the section of method. We can clearly see that if *g* = 0, 
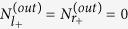
, and 
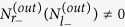
, which means that without the mechanical oscillator we only have two-port router, and the optomechanical coupling is necessary for us to realize multi-port router.

**Figure 3:**
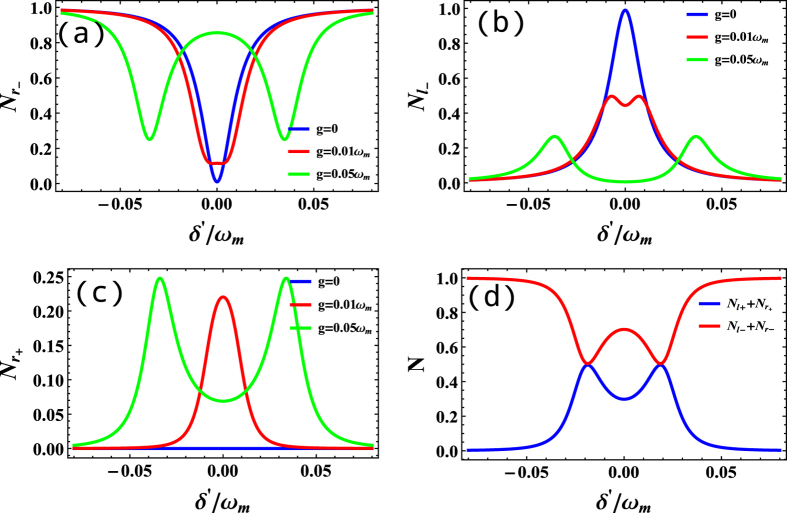
(**a**–**c**) Photon number *N*_*r*−_, *N*_*l*−_, 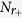 as function of *δ*′ for several values of *g* where *γ* = 0.01*ω*_*m*_. (**d**) 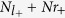 and 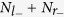 as function of *δ*′ with *g* = 0.04*ω*_*m*_. It is naturally satisfied normalized condition 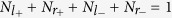. And *ε* = 0.0001*ω*_*m*_ all the parameters were normalized by *ω*_*m*_.

We plot the output photon number of the four ports as a function of *δ*′ for several values of *g* in Fig. 3(a–c). If *g* = 0 (means without the coupling of radiation pressure), when *δ*′ = 0 (*δ* = Δ_−_ denotes that the input photon is on resonant with the cavity fields), the single photon will almost transmit into the left port 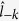 which was equivalent to a common cavity waveguide coupled system which present a perfect reflection at resonance region and only one peaks (valleys) with linewidth 2*γ* showing in blue line of Fig. 3(a,b). With the increasing of *g*, the photon will be partially transmitted and partially be reflected, but they are still of one peak (valley). However, with the increasing the values of *g*, for example *g* = 0.05*ω*_*m*_, the single peak (valley) is split into two peaks (valleys) because the movable mirror participates the three-body interaction so that we can see the symmetry peaks (valleys). Most importantly, the one port input signal can be distributed into four ports see Fig. 3(a–c), while for *g* = 0, we can receive only two ports signals 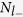 and 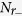. Therefore, with the assistant of the two coupled cavity optomechanical system, we can realize multi-port router. We parcel the four-port output into two parts 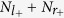, 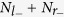 because they denote the difference whether the optomechanical coupling is included or not, seeing Fig. 3(d). Though we can transport the photon via the optomechanical coupling, the probability of transportation 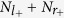 is still less than 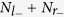 under the group of the parameters, which means that the optomechanical coupling constant g strong affects the router process.

**Figure 4:**
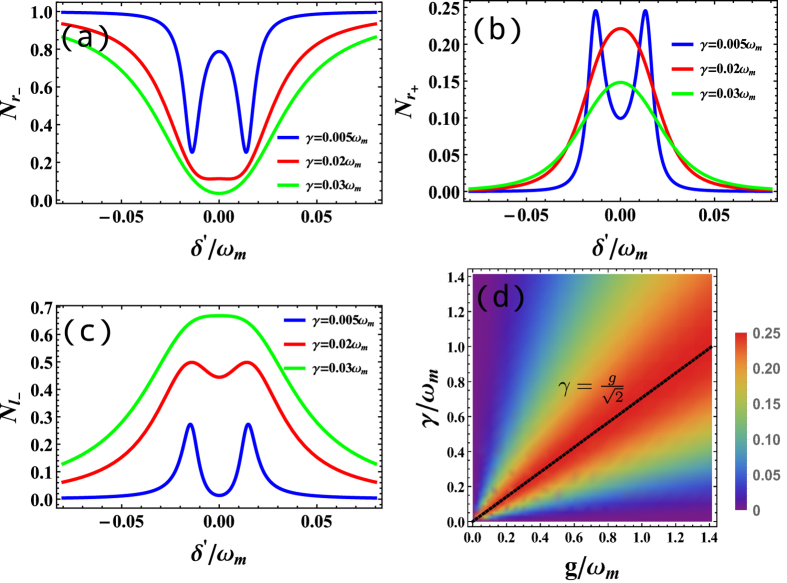
(**a**–**c**) Photon number *N*_*r*−_, *N*_*l*−_, 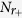 as function of *δ*′ for several values of *γ* = 0.005*ω*_*m*_, 0.02*ω*_*m*_, 0.03*ω*_*m*_ represented by blue, red, green line respectively, where *g* = 0.02*ω*_*m*_. (**d**) Photon number 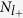 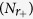 versus *γ* and *g* when *δ*′ = 0. The dash black line highlight the maximum of output. *G*_1_ is a normalization coefficient to guarantee 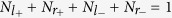, *ε* = 0.0001*ω*_*m*_ all the parameters were normalized by *ω*_*m*_.

Besides the optomechanical interaction, the cavity-fiber coupling should also have important influence on the router process. As shown in Fig. 4(a–c), we plot the multi-port output photon number under the same optomechanical coupling *g* = 0.02*ω*_*m*_ but with different cavity-fiber coupling constants *γ*. For *γ* = 0.05*ω*_*m*_, we can observe the split of peaks (valleys). However, with the increasing of *γ*, even with the same optomechanical interaction *g*, one only can see single peak (valley), which means that strong cavity-fiber coupling can suppress the function of optomechanical coupling. That is to say, there is a competitive relation between and cavity-fiber interaction and the optomechanical coupling. In order to make clear the match relation, we plot optimized 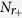 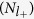 as function of the parameters *g* and *γ*, shown in Fig. 4(d). We observe that when there is an optimized value 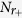 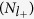 along the line 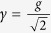, which exhibit that the balance between cavity waveguide coupling and optomechanical interaction is helpful to the multi-port router procession.

## Conclusion

We put forward a scheme to realize multi-port router using two coupled cavity optomechanical system. We first demonstrate that our system with the Hamiltonian Eq. (8) can be effectively equal to the three-body interaction between cavities and the oscillator which has been shown in ref. 35. The nonlinearity in the three-mode mixing is not proportion to *g*^2^/*ω*_*m*_ and can overcome the restraint 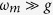. We also numerically show the nonlinearity and correction of the effective interaction. By coupling the two coupled cavity optomechanical system to waveguide, we calculate the output photon number of the multi-port router. Our results show that the presented system can work as multi-port router under the witness of the optomechanical coupling. Since the two coupled optomechanical cavity is similar with the experiment^36^ where the optomechanical coupling is ignored. If the optomechanical coupling is strong enough, our scheme should be realizable.

## Methods

### Router

Now we solve the Schrodinger equation of this system with Hamiltonian 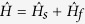 and wave function Eq. (12).


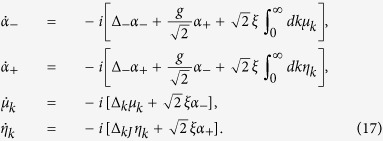


We assume that initially the cavity is in the vacuum state, and a single photon with the waveguide, i.e., |0, 0, 0, 1_*k*_, 0〉 is prepared in a wave packet with a Lorentzian spectrum, the initial condition reads 
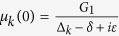
. Using Laplace transformation, the differential equations Eq. (17) become


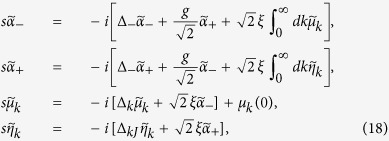


In the long-time limit, the coefficients *μ*_*k*_(∞) and *η*_*k*_(∞) are obtained after inverse Laplace transformation as


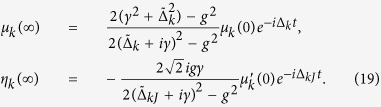


where *γ* = 2*πξ*^2^ denoting the cavities loss into the waveguide. If there is no the other decay except the exchange between the cavities and the waveguide, *γ* will be equal to the decay rate of the cavity which we have mentioned in Fig. 2. In terms of the left- and right-propagation modes, if we assume a photon packet is incident onto the cavity from the port *r*_−*k*_, then the initial state can be written as


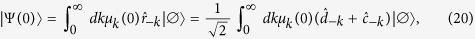


which means that the single photon input from the port *r*_−*k*_ can be considered as a superposition between a quasiparticle 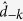 and a quasiparticle 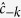. In the long-time limit, the wave function becomes under the Hamiltonian Eqs (8) and (9)


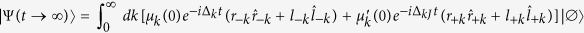


where the first bracket with the factor 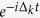 can survive without Hamiltonian Eq. (8), while the second bracket with the factor 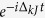 survive only under the condition Eq. (8) existence. In other words, the photon on the ports *r*_−*k*_ and *l*_−*k*_ can be detected even without the mechanical mode, however, if we would like to obtain photon on the port *r*_+*k*_ and *l*_+*k*_, the coupling between the mechanical mode and cavity field is necessary. Then we obtain


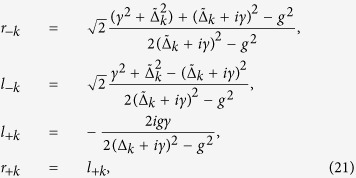


and the output photon number in Eqs (14), (15) and (16).

## Additional Information

**How to cite this article**: Li, X. *et al*. Single-photon multi-ports router based on the coupled cavity optomechanical system. *Sci. Rep.* **6**, 39343; doi: 10.1038/srep39343 (2016).

**Publisher's note:** Springer Nature remains neutral with regard to jurisdictional claims in published maps and institutional affiliations.
